# Some of the Drug Conditions during the War between the States, 1861-65

**Published:** 1898-10

**Authors:** Jos. Jacobs


### SOME OF THE DRUG CONDITIONS DURING THE WAR BETWEEN THE STATES, 1861-65.

BY JOS. JACOBS.

Here, in grand old Maryland, this border State of the bygone Confederacy, at a time when men of that war generation who fought on either side of a great and memorable conflict meet with the sons of both in friendly conference, at a time and place where none can be stirred to animosities by recalling the subject, I present a paper relating to the drug trade and drug conditions as they appeared during the War of 1861–65, especially as they existed in the Southern States.

Whatever may be the final verdict of mankind as to the justice of the cause for which the seceding States engaged in warfare with their kindred commonwealths, it must follow the recorded admission of the heroism and magnanimity of the Southern people in maintaining that brave struggle in arms against the proud and wealthier section of our common country; and, just as sure as that now the old soldiers of the South and their sons stand as ready to answer any call of our splendid Union of States against any and every foe, as the old soldiers of the North and their sons, just as sure are the hearts of all willing to still all sentiments in reference to the old conflict of arms, excepting such as spring from pride in the valor of those who wore the blue and those who wore the gray.

There are few American citizens, to-day, who would not rejoice if the bloody record of that war, with its story of suffering and death, had no place in our history. Would that we, as brother Americans, had never been compelled to witness any of the scences or consequences of that sad conflict, and that our children should never have been called upon to turn the pages of its annals.

We can not doubt the existence of genuine reconciliation now, since the calls that have so recently assembled our gallant boys from every State in our Union, and who, mingled together, have illustrated the common valor of Americans in arms against the Spanish hosts, and whose acts of heroism are now recorded in never-dying lines that shall commemorate the worth of North and South, and East and West, alike.

As pharmacists, rejoicing in the existence of a truly re-united country, we should recognize that we must ever stand ready to do our part should foemen ever invade our territory, standing true and firm though we should be isolated from all the nations of the earth; and so, looking back over the days of the war between the States, I have endeavored to see if there were not some lessons to be learned from the adversities in which the Southern people found themselves in the matters that particularly relate to our profession. For, when a people is put to straits and when overwhelming necessities confront them, invention is stimulated, experiment prompted, and out of their very helplessness, often, intelligence is aroused and action follows which evolves new and valuable accomplishment.

The Southern people prior to the war were almost exclusively an agricultural people. The broad acres of the South yearly whitened in fleecy cotton, or waved with yellow grain, or sent forth from their soil the cane and rice harvests, or pastured the flock within their confines. At the beginning of the war, except at Richmond and a few other of the more northerly cities, there were very few machinery plants, and the factories and foundries which produced articles of cotton or wool or brass or iron and steel, were small in number and in the extent and variety of their productiveness. The splendid water-powers of the Carolinas and of Georgia that now mingle the music of their falling with the hum and whirr of textile mills, wasted over their rocks as they ran to the sea by the cotton fields in the broad, alluvial valleys. Boats that ran up the Mississippi and the Ohio were laden with the cotton and wheat of Texas and the sugar and syrup of Louisiana or the imported products of the Mexican Gulf countries, and they returned freighted with coal and iron and all the varied manufactured products of the North and East. Tennessee, Arkansas, North Carolina and Virginia were dotted with granaries and tobacco barns, and sent their "cattle" from a "thousand hills" into the markets of the country. Florida and Mississippi were largely engaged, besides in the production of the usual Southern crops, in furnishing the fruits of their orchards and the output of their fisheries to commerce The inexhaustible beds of iron ore and manganese and coal of Georgia and Alabama and Tennessee were still unexplored, and the vast quarries of Georgia marble and granite, now yielding rich profits to Northern investments, were then overlooked and unworked.

It can be imagined that a territory like this, unprepared for war and sustaining an ignorant slave population which amounted to at least two-fifths of the whole number of persons, suddenly confronted by an armed conflict and at once invested by vigorous, watchful and competent blockading fleets, full of natural resources, deficient in organized industries, rich in the possession of men of intellect and executive capacity, would be met by a situation calling forth every talent and resource of its people.

Side by side in the columns of the newspapers, with the stirring appeals to patriotism in editorial language and poetic meter, were official orders and advertisements; and scientific and literary men vied with one another in publishing suggestions and hints and descriptions of processes that would be useful in directing the minds of the people toward solving the problems of supplying necessary munitions of war and all the articles for camp and field and hospital and household use.

To say nothing of the destruction of property and of the whole labor system of the South with its attendant losses, some idea of the extent of the effects of that war may be gathered by reciting a few facts from official data.

Eleven out of the thirty-four States seceded. The men of military age from eighteen to forty-five on the Southern side numbered 1,064,193, including lame, halt and blind, etc. On the Union side were more than four to one, or 4,559,892, not estimating monthly accessions from the world at large. In enlisted men the numbers were, for the South, 600,000; for the North, 2,865,000. The slave States of Kentucky, Missouri, Maryland, West Virginia and Tennessee gave to the Union 300,000 men. Thus there were in the field four armies of the North, each as large as the entire Confederate forces, not including the 300,000 men contributed by the slave States.

In numbers the Federal loss was 67,058 killed and 43,012 died of wounds; of Confederates, 53,873 were killed, and 194,026 was the number of killed and wounded on the fields of battle. More than one-third of the Confederates were confided to the surgeons, besides the sick and wounded prisoners of war.

The Confederate government, immediately after the formation of a provisional government at Montgomery, were confronted by strong facts and large figures as to supplies for the different departments. Agents were sent at once to Europe, most of whom were in London, and where they established a weekly newspaper, with local correspondents in nearly every Southern town from Virginia to Texas. Instructions were given that, as there were only two sources of supply, capture and blockade running, importance was to be given to securing first, arms and ammunition; second, clothing, including boots, shoes and hats; third, drugs and chemicals, such as were most pressingly needed, as quinine, chloroform, ether, opium, morphine, rhubarb, etc. These agents were instructed to see that all blockade runners or any transport ships, barks or brigantines, that were clearing for Southern ports for cargoes of cotton or naval stores, were loaded with the above enumerated articles; the cargoes to be consigned to individuals, firms or agents of the government at any port to which they cleared.

At the outset of the struggle the question of drugs and medicines was thus third in importance, and the druggists of the South had either to manufacture what they could from native barks and leaves and herbs and roots, or purchase at Southern ports such supplies as the blockade runners brought in that were not intended for the government. In most cases these cargoes were offered at auction. This was a custom at Galveston, New Orleans, Mobile, Charleston, Pensacola, Savannah and Wilmington. The gulf cities received large supplies from Cuba; while in Texas there was almost a continuous train of contrabanders, or smugglers, bringing goods across the Rio Grande from Mexico, but not much of this was medicine.

As to capture, while the army frequently captured the wagon trains of the enemy, thus obtaining some supplies of medicines and surgical appliances, these were barely sufficient to supply the most distressing needs in the army; so it may be seen that home manufacture and blockade running were the only source of supply during nearly four years for between six and seven millions of people.

The interior towns suffered most; such places as Jackson, Meridian, Columbus and Aberdeen, in Mississippi; Selma, Montgomery, Eufaula, Huntsville, in Alabama; Albany, Columbus, Macon, Augusta, Athens, Rome and Atlanta, in Georgia; Spartanburg, Greenville and Columbia, in South Carolina; Fayetteville, Goldsboro, Raleigh, Statesville and Charlotte, in North Carolina; and Danville, Lynchburg, Petersburg and Richmond, in Virginia. In nearly all of these towns one or more druggists manufactured from stock on hand of roots, herbs and barks, or from home supply of such medicinal plants as he could secure, tinctures and like preparations.

The supply of whisky was not so short as that of medicines. The so-called "moonshiners" of the mountains of North Carolina, Tennessee, Alabama and Georgia kept their stills (often made of gum logs) running night and day, and could find a ready sale for all they produced. So far as I can learn, no tax was placed on whisky. In New Orleans rum was made from molasses, one distillery turning out over one hundred barrels of this product every day for over a year.

Amongst the scarcest articles in a drug store in those days were paper, twine and corks. Some of the stores obtained old life-preservers fom abandoned river boats and got a supply, thus, of hand-cut stoppers. Various fabrics were pressed together for small stoppers, and for large bottles, demijohns and jugs, different sized corn-cobs commanded the same price as XXX corks do to-day. In the museums of New York, Washington and Chicago can be seen some of the specimens of attempts to manufacture glass bottles in Louisiana, Alabama and South Carolina.

In the interior districts and small villages the country doctors returned to first principles and to the use of the plants of the fields and forests; and these agencies were about all they had to rely on, outside of whisky and a little quinine, the latter frequently at $100 an ounce.

Interviewing one of our old Confederate surgeons, he said:

"During the early part of the war I was placed in charge of a railroad hospital in a small town where it was difficult to obtain medicine at almost any cost, and as I had my little hospital crowded nearly all the time, both with employes of the road and wounded and sick soldiers, afflicted with various diseases and all kinds of wounds and injuries, and being also engaged in general practice, it naturally followed that my mind was severely taxed in order to supply remedies and substitutes to meet the demands of such varied practice. I perused my dispensary and called into requisition an old botanic practice that had been handed down as a relic of the past, but from which I confess to have received valuable aid and very many useful hints in regard to the medical virtues of our native plants. I give you the following facts from a record I kept of the patients treated, and the remedies I used, and the principal substances I resorted to.

"Of that large class of medicines, so useful in surgery and so much in demand in war times, called antiseptics, most of them, I may say, have been discovered and appropriated to surgical use since our war. In fact, I had but little else at my command except the cold-water dressing for wounds. From experiment I learned to improve on the plain old method, as I think, by employing a decoction of red-oak bark added to the water, which acted as a disinfectant, and by its stimulating and astringent properties promoted the healing process. I also used a weak solution of bicarbonate of soda, which I found beneficial in the suppurative stages. When emollients were indicated, I used slippery elm and wahoo root bark, and solution of common salt often helped. In case of great pain I employed poppy heads, nightshade and stramonium.

"I had a number of cases of intermittent fever. I would give strong boneset tea, warm, until free vomiting was produced, and as a substitute for quinine I used, during the intermissions, butterfly root or pleurisy root tea, which would nearly always cut short the febrile stage.

"Remittent or bilious fevers were treated much the same way, except that I invariably gave good doses of mandrake tea in the beginning. White ash root or prickly ash root were often given in these fevers to advantage, using always the butterfly-root tea in the febrile stage. Virginia snake-root, yellow root, or Sampson's snake-root acted nearly as well, but I preferred the other. If I could have obtained blue mass or calomel I would have begun treatment with a dose or two of that, but rone were to be had.

"May-apple root or peach-tree leaves made into a strong tea and drank warm would act on the bowels as certainly as senna; but with children, where too much tea is not desirable, I often gave beef's feet oil, hog's feet oil, or even lard heated with syrup.

"In cases of pneumonia, pleurisy, catarrhal fevers, etc., I made local applications of mustard seed or leaves, stramonium leaves, hickory leaves, pepper, etc., warm, and gave alternately butterfly-root and sanguinaria, and continued to slightly nauseating, from day to day (no need of anything else). The two last-named remedies took the place of Dover's powder, quinine and all other diaphoretics, febrifuges and arterial sedatives.

"Phytolacca or poke was another favorite remedy—the tincture, when alcohol or whisky could be obtained; otherwise, tea of root or berries. I used it in all cases of chronic rheumatism or neuralgia, enlarged glands, scrofula, syphilis, and all cases requiring alteratives, often combined with American sarsaparilla root, sassafras, alder and prickly ash.

"Female complaints gave me some trouble, but I soon learned the use of black haw, squaw-weed, partridge berry, etc. I had been taught in the old text-books that opiates in large doses would control some cases of threatened abortion, when the patient had not lost too much from hemorrhage. I found that the black haw root tea would absolutely stop this tendency, not only in cases where there was but little hemorrhage, but where large quantities had passed, and would relieve the most severe case of dysmenorrhea, especially when combined with squaw-weed, partridge berry or red shank.

"In stomach and bowel diseases I found but little difficulty in obtaining plenty of substitutes for opiates, astringents and the like; in fact, I believe that an all-wise Providence has especially provided the best antidotes in creation on the hills and dales and by the vales and streams of our own Southland. In ordinary looseness of bowels or diarrhea I gave an infusion of raspberry leaves or whortleberry leaves (both of which act finely on the kidneys and bladder). Where there was nausea or sick stomach, a handful of peach-tree leaves steeped in water and drank will settle it, or what is perhaps better, the kernels of two or three seeds cracked and cold water drank off of them. If stronger astringent is necessary, the inner bark of red oak, blackberry or dewberry root tea, or red shank root, are sure remedies.

"Agrimony tea, and, as a last resort, the nut-gall or ink-ball made into what, from its color, I called black wash (made by squeezing the juice out and adding a little copperas). This black wash is not only a splendid ink, but is a destroyer of syphilitic sores, warts, corns, ringworm, and old ulcers and excresences of nearly every kind, much superior to lime-water and calomel. Weakened properly, it is good in obstinate bowel diseases, and can be used as an injection in gonorrhea, gleet, etc. Silk weed root put in whisky and drank, giving at the same time pills of rosin from the pine tree, with very small pieces of blue vitriol, will cure obstinate cases of gonorrhea, and is a substitute for copaiba, cubebs, etc.

"I raised lobelia from the seed, and found it to be a reliable emetic, useful in cough medicines, croup and asthma. I have relieved asthma with lobelia and by smoking stramonium leaves. We, of course, used turpentine as an adjunct in all cases where indicated, which is the case in very many diseases and in many a positive curative agent.

"Onions and garlic were useful as poultices in nearly all glandular enlargements, as are also poke-root, celery, pepper, parsley, sage, thyme, rue and other garden products. Many of the latter were used for diseases of women and children.

"White sumac, red elm, prickly ash, and poke, will in connection with my black wash cure recent cases of syphilis. It will also cure many cases of chronic rheumatism. Peach-tree leaves and Sampson's snake-root will cure most cases of incipient dyspepsia. Gargle made of sage and honey will cure most cases of sore throat, tonsilitis, etc.

"For infants, calamus, catnip and soot teas are better than soothing syrup with opiates."

Nearly every old practitioner in the South, to day, is full of such reminiscences as the foregoing.

Notwithstanding the restrictions on inter-state commerce and the almost exclusive reliance on blockade runners for supplies, many druggists in these Southern towns and cities displayed much ingenuity in the disposition of the stocks bought at auction at the seaports.

Mr. H. Metcalf, of Montgomery, relates that he attended an auction sale at Mobile on one occasion, and, arriving late, found the cargo all sold except cod-liver oil and beeswax, which he succeeded in purchasing. His two barrels of cod-liver oil and 600 pounds of beeswax were immediately reshipped to Montgomery on the Alabama river. Filling every shape and size bottle to be found, and placing a judicious advertisement in the papers, he was enabled to sell the oil, but what to do with the beeswax was a puzzler. Discovering a set of candle-moulds and using cotton yarn as a wick, he ran the entire mass into candles and succeeded in selling the whole stock at ten cents a piece.

Nashville fell early in the action, and there was but little suffering there on account of failure to obtain medical supplies. One incident is related there showing the shrewdness of druggists at Nashville. When it became known that all the manufacturing enterprises would be blown up on the evacuation of the town, a shrewd druggist went around and succeeded in buying all the window glass in town. Three days later the explosions, on the retreat of the Confederates, broke one-half the window glass in the city, and Mr. S. reaped a rich harvest from his corner in window glass.

Various small attempts were made to manufacture chemicals at Knoxville, Tenn., Greenville, S. C., Columbia, S. C.,

Milledgeville and Macon, Ga., but, outside of producing a few gun-caps and nitre for making gunpowder and a few carboys of sulphuric acid for charging the torpedoes in Charleston harbor, very little was accomplished. Later on some small manufacturing was done at Richmond and Charlotte, but, owing to the want of machinery and proper apparatus, little was achieved. A blockade runner brought into Wilmington, N. C., a supply of apparatus for making sulphuric acid, which arrived only a few days before the city fell. Much might have been accomplished with this but for the fall of Wilmington, as the plant was said to be first class, and, it is said, was disposed of for a large sum to a Philadelphia manufacturer.

The excessive high price of quinine made its handling a profitable employment. Almost every means known to human ingenuity was employed to smuggle it through the lines. Small packages were placed in letters, which the Adams Express Company would guarantee for the sum of $2.00 to deliver to the post-office authorities at some point in the Confederacy. Officers speculated in it, buying and selling until this created a scandal almost equal to that of speculating in cotton, and it was finally stopped by a strong proclamation.

A large contraband trade was carried on by an almost continuous line of house-boats floating on the Mississippi river. When opposite Memphis the goods were either sent in at night or into the interior of Arkansas, where trusty parties soon disposed of the stock. The great bulk of this trade was sent out by traders and speculators in Paducah, Ky., and Cairo, Ill., and their main points of operation were Memphis, Tenn., Helena, Ark., Napoleon, Ark., and Greenville, Miss. In regard to Napoleon, very few of this generation ever heard of the town, nor can it be found on the maps of the present day; yet in war time Napoleon, Arkansas, was a town of nearly 3,000 people, well built with brick business houses, and contained a large United States marine hospital, built of brick; and situated as it was on the Mississippi, at the mouth of the Arkansas river, it was at one time a rival of Memphis for trade. This village was entirely destroyed by flood in 1869 or 1870; the last vestige of the large marine hospital was carried into the Mississippi river in 1874, and to-day there is not a human habitation left to show where Napoleon once flourished.

One of my Alabama lawyer friends, an ex-Confederate famous for learning, for valor as a soldier and for delightful humor as a raconteur, once related to me the following reminiscences:

To supply the trying necessities of the drug demand, he said he had heard of many amusing plans that were resorted to by the government itself, and by persons who were mainly prompted by neither impulses of humanity nor patriotism, but by the simple desire of gain. He said he heard of a woman who went into the Northern lines four times, returning always with a considerable quantity of the more costly drugs concealed beneath her skirts. On her return from the fifth trip, however, some portion of her paraphernalia, while on a ferry boat, was caught in a way to put too great a strain on some string or buckle, so that it gave way, and the walking drugstore was brought down to "dire combustion."

A Mr. Berg, a merchant of Middle Alabama, says my Alabama friend, at the beginning of the war found himself with empty shelves and counters and no market from which to replenish his stock. He had had some experience in the sale of drugs and medicines, so he determined to occupy his genius, being too old to go to the war, by carrying on a contraband trade in this profitable direction. He started on a dangerous enterprise, as the South had interdicted trade in cotton and the North had placed the ban on drugs—especially on stimulating liquors. Mr. Berg selected Memphis as the base of his operations, and proceeded up to the northern part of Mississippi, a country alternately in the hands of the Confederates and the Federals. Here he purchased a common road wagon and four mules, and loaded the wagon with cotton. In a few days he arrived, with an assistant, within the Federal lines at Memphis, where he disposed of his cotton at war figures, for United States money. His wagoner, having received his reward, deserted, and Berg could find no one to go back with him to the South. He was about to abandon his enterprise of investing in drugs and medicines for lack of proper means of transportation, when he accidently, while looking after his own team and wagon, discoverd a two-horse vehicle, considerably battered and disfigured, but surmounted by a white cloth covering, over which was a small yellow hospital flag, and upon the sides of which was painted in large letters "Small-Pox." In a short time Berg had exchanged his four-horse vehicle for the smaller one, and selecting two of his best mules, hit upon the idea of transposing his hospital wagon into a blockade runner. He soon had a stock of quinine, morphine, ether and such other drugs as promised the greatest profit stored away in a box under the yellow flag, and over these he placed several layers of the leather fronts for making cotton and wool cards, over these some cheap clothing, and as a last layer scattered promiscuously a collection of such articles as are usually carried in a peddler's pack, including cambric needles. The enterprise might have been entirely successful had not Berg determined to add to his stock an eight-gallon keg of good rye whisky, then exceedingly scarce in his native region.

Berg proceeded on his return journey very slowly. The roads were bad, his team weak, and he inexperienced. The yellow flag above his wagon and the legend on its sides accomplished fully all he had expected from them, so far as keeping him unmolested and preventing his contraband cargo from being detected. They were equal to the ancient cry, "Make way for the leper."

Berg himself also grew quite travel-stained, and to ordinary observation had but recently recovered from the small-pox. The end of the fourth day found his stock of provisions, both for man and beast, entirely exhausted, while every attempt on his part to approach a farm-house in order to obtain these necessities was met with threats and the barking of dogs, and he and his team went into a supperless camp. The next morning he concealed himself some distance from the highway, tied his mules out in a swamp to graze, and, having scrubbed himself up in a neighboring stream, started out afoot in hope of finding some farm-house remote from the highway where he might negotiate for provisions. Before starting, however, in order to fortify himself against the fatigue of the journey, Berg for the first time uncovered his hidden keg and drew off a bottle of its costly contents, drinking some of it before starting. An hour's wandering brought him at last to a farm which gave promise of creature comfort and refreshment. There was a woman in possession of the house as Berg approached, who forbade his coming any nearer than the gate, firmly and positively denying all his entreaties to save him from starvation. At last, however, she told Berg, who had so far forced his way into her presence that she detected the smell of whisky, that if he would furnish her a bottle of that article she would, in exchange, give him food for himself and his mules; and, as this was the only alternative, the bargain was made and she went to work preparing the provisions, while Berg returned to the wagon with the bottle she furnished. Berg had just finished his chicken and onions and bread, and the mules disposed of their fodder, and everything was in readiness for the journey to be renewed, when, with shouts and clattering hoofs, four blue-coated troopers rode up. In some way they had gotten hold of the whisky from the woman and learned from her the source of supply, and had tracked Berg to his camp. They had drank enough of the whisky to render them utterly indifferent to death or contagion in any form, and, while Berg was swearing he had no whisky, they were prying into the wagon and were emptying the keg through its bung hole into their tin-cups as freely as if it were branch water; and then they began to torment poor Berg with all manner of pranks and tricks. Finally, one of them determined to make him swallow a paper of the cambric needles, and had actually placed them on his tongue, handing him a cup of his own whisky and threatening to cut him down with their swords unless he swallowed the needles with a draught of the whisky.

Berg said that at that momemt he lost consciousness, and did not ever know whether he swallowed the needles or not; that when he awoke a man was bending over him asking what was the matter with him. The shouts of the drunken soldiers had attracted the attention of a party of Confederates, who, coming up unawares, had killed two of Berg's tormentors and wounded one severely, allowing only one to escape.

In such conditions as these, it is not to be wondered at that every kind of makeshift and substitution had to be resorted to in the field, in the drug store, and upon the farms and in the household.

Many times the Confederate soldier marched and camped and fought on half rations. The full ration was meagre enough. As prescribed, it was as follows: ¾ lb. of pork or bacon, or 1¼ lbs. fresh beef; 18 oz. bread or flour, or 1¼ lbs. corn meal. On campaigns or marches or on transports the ration of hard bread was one pound.

The following will give an idea of the economy that was enjoined in the matter of supplying general and post hospitals, the amounts stated being quantities for one year for one thousand troops: Acetic acid, 5 lbs.; arsenic, 5 ozs.; muriatic acid, 8 lbs.; suphuric acid, 8 lbs.; tartaric acid, 16 lbs.; sulphuric ether, 16 lbs.; alcohol, 192 pint bottles; ammonia, 5 lbs ; nitrate of silver, 8 ozs. assafœtida, 32 ozs.; camphor, 16 lbs.; catechu, 5 lbs.: ceræ albæ, 16 lbs.; chloroform, 8 lbs.; copaiba, 40 lbs.; creosote, 16 ozs.; adhesive plaster, 40 yards; extract belladonna, 16 ozs.; fluid buchu, 8 lbs.; columbæ, 8 lbs.; gentian, 8 lbs.; glycyrrhiza, 48 lbs.; hyoscyani, 16 ozs.; rhei, 8 lbs.; sarsaparilla, 16 lbs.; senna, 8 lbs.; valerian, 64 ozs.; mercuric chloride, 5 ozs.; iodine, 16 ozs.; ammonia, 32 lbs.; magnesia, 5 lbs; sulphate morphia, 16 drs.; myrrh, 5 lbs.; opium, 5 lbs.; ether, 5 lbs; jalap, 32 ozs.; cantharides, 16 ozs.; aloes, 32 ozs.; sulphate quinine, 80 to 160 ozs.; sugar, 160 lbs.; strychnia, 8 drs.; digitalis, 32 ozs.; unguenti hydrarg., 8 lbs.

The same sparse quantities were applicable in hospital stores' regulations and in the matter of surgical instruments, books, bedding, furniture. dressings, etc., and on the blanks furnished was printed the following: "It is urged that medical officers make requisition for such medicines only in the following table as are deemed *indispensable.*"

Dr. J. Julian Chisholm, professor of surgery in the Medical College of South Carolina, published in 1861 his "Manual of Military Surgery for the Use of the Surgeons in the Confederate Army." This book was widely used, and was a most valuable contribution to war surgery, containing, as it does, a most exhaustive collection of hints and instructions relative to the treatment of sick or wounded men in camp, on the field of battle and in the hospital. In his preface he says (in part)

as follows: "As our entire army is made up of volunteers from every walk of life, so we find the surgical staff of the army composed of physicians without surgical experience. Most of those who compose the staff were general practitioners, whose country circuit gave them but little surgery and seldom presented a gun-shot wound. Moreover, as our country had been enjoying an uninterrupted state of peace, the collecting of large bodies of men and retaining them in health, or the hygiene of armies, had been a study without an object and therefore of little interest."

From my friend, J. F. B. Lillard, of New York, I learn the following names of some druggists who were in business at the South during those trying times: Benjamin Ward, of Mobile; H. Metcalf, at Montgomery, Ala.; J. A. Lee, New Iberia, La.; N. O. Mior, Columbia, S. C.; Jno. Ingalls, Macon, Ga.; J. J. Shott, Galveston, Tex.; F. S. Duffy, Newbern, S. C.; G. W. Aymer, Charleston, S. C.; S. T. Dernoville and A. H. Roscoe, Nashville, Tenn.; Robert Carter, Columbus, Ga.; A. Solomons, Savannah; Ga., Crawford W. Long, Athens, Ga.

To afford an idea of the prices ruling in Richmond, June, 1863, I append the articles in some original invoices purchased by R. W. Powers from Kent, Paine & Co. Some are as follows: 3 boxes ext. logwood, 47 ℔s. at $4.00 per ℔.; 1 keg bicarb, soda, 112 ℔s. at $2.75; 1 case brown Windsor soap, $12.75 doz.; 1 bbl. camphor, 86 ℔s., at $20.00; 112 ℔s. of blue galls at $4.00; 100 ℔s. tartaric acid, $2.25 per ℔.; salt, 44 cts. ℔.; hops, $2.50 ℔.; 1 cask French brandy, $52.00 gal.; Indian ink, 75 cts. bottle; 9 doz. assorted pencils, $4.00 doz.; phosphorus, $14.00 ℔.; citric acid, $4.50; oil peppermint, $16.50; Epsom salts, $3.87½; 6 bottles capsules, $6.50; 12 pewter syringes, $1.25 each; 2-box blue pills, $6.00; 1 bottle syr. ipecac, $10.00; 15 ozs. quinine, $22.25 oz.; 60 dr. morphine, $28.00 dr.; blacking, $1.40 per box; tallow candles, $2.37 ℔.

H. B. Metcalf, of Montgomery, wrote me in February last in part as follows:

"I find that all my old books and papers were destroyed in the fire of last July. We were able to secure some drugs and chemicals during the war by attending the blockade sales at Charleston and Mobile. We did not have to substitute to a great extent in putting up prescriptions—those of us who were fortunate enough to be supplied at the sales. We found great difficulty in securing vials and corks, and were compelled to use second-hand vials, and corks made from the tupelo tree answered very well. Prices were, of course, high; for instance, during the last year of the war all tinctures were sold at $1.00 an oz.; quinine, $25.00 per oz.; morphine, $10.00 per dr.; quinine pills, $1.00 each, and other pills $5.00 a dozen. Prescriptions ranged usually from $5.00 to $15.00. Whisky sold at $150.00 a bottle. You must recollect that greenbacks were worth about twenty times our money, gold 100 times. I imported a great many goods through Evans' Sons, Liverpool, and regret exceedingly I now have none of the invoices."

It was quite an industry, I am told by an Atlanta lady, Mrs. Marcus A. Bell, for the country people to raise castor oil beans. The crushed beans were boiled and the oil skimmed off. She said that the grandmothers of those days revived the traditions of Colonial times. They made their own dyes and coloring matters from the roots and barks of native woods. Dogwood, sumac and the roots of pine trees were largely used, and indigo was cultivated in the gardens. Instead of paregoric, fennel-seed tea was given to the babies. For rash they used red-oak bark and alum. Goose grease and sorghum, or honey, was a standard remedy for croup, backed up with turpentine and brown sugar. Sassafras tea was given in the spring and fall as a blood medicine. Adults' colds were doctored with horsemint tea and tea from the roots of broom sedge. For irruptions and impure blood, spice-wood tea was given. Wine was made from the berries of the elder bush. For diarrhœa, roots of blackberry and blackberry cordial; and so, also, was a tea made from the leaves of the rose geranium. Mutton suet, sweet gum and the buds of the balm of Gilead was a standard salve for all cuts and sores. Balsam cucumber was widely used as a tonic, and was considered a specific remedy in burns. Catnip, elecampane, and comfrey root and pennyroyal were in every good housewife's pantry, in which, also, was the indispensable string of red peppers, a bag of sage leaves and of "balm." Calamus root for colic in babies was a common dose. The best known standard Georgia tonic was dogwood, poplar and wild cherry barks, equal proportions, chipped fine and put in whisky and taken wineglassful at meal times; it is still used in large quantities from "Yamacraw to Nickajack." In hemorrhages, black haw root was commonly used. All the white mustard we had was raised in our gardens.

She learned from these experiences that barks were best gathered while the sap was running, and when gathered the outer and rougher portion should be shaved off and the bark cut thinly and placed in a good position in the shade to dry; that roots ought to be gathered after the leaves are dead in the fall, or better, before the sap rises; that seeds and flowers must be gathered only when fully ripe, and put in a nice dry place, and that medicinal plants to be secured in the greatest perfection should be obtained when in bloom and carefully dried in the shade.

I here append a list of substitutes that were used by druggists and physicians during the war in large quantities, in most of the instances being the only medicines of the kind to be had.

| IMPORTED ARTICLE. | SUBSTITUTE. |
|---|---|
| *Columbo Quassia* | Yellow root. |
| *Spanish Flies* | Potato bugs. |
| | Powdered leaves of butternut. |
| *Jalap* | Wild jalap. |
| | Wild potato vine. |
| | Fever root. |
| *Aloes* | Wild jalap. |
| | Mulberry bark. |
| | Butternut. |
| | Dock. |
| | Wild potato vine. |
| | Amer. colombo. |
| *Quinine and Peruvian Bark* | Tulip-tree bark. |
| | Dogwood. |
| | Cotton-seed tea. |
| | Chestnut root and bark. |
| | Chinquepin root and bark. |
| | Thoroughwort. |
| | Spanish oak bark. |
| | Knob grass. |
| | Willow bark. |
| *Digitalis* | Blood root. |
| | Wild cherry. |
| | Pipsissiwa. |
| | Bugle weed. |
| | Jasmine. |
| *Conium* | American hemlock. |
| *Opium* | American hemlock. |
| | Mother-wort. |
| *Sarsaparilla* | Wild sarsaparilla. |
| | Soapwort. |
| | Yellow parilla. |
| | China briar. |
| | Queen's delight. |
| *Chamomile* | Dogwood. |
| *Flaxseed* | Watermelon seed. |
| *Gum Arabic* | Low mallows. |
| | Apple, pear and quince gum. |
| | Balm. |
| | Watermelon seed. |
| *Ergot* | Cotton-root. |
| *Guaiacum* | Boxwood. |
| | Poke. |
| | Prickly ash. |
| *Ipecac* | Wild jalap. |
| | Carolina hipps. |
| *Mezereon* | Prickly ash. |
| *Kino and Catechu* | Cranesbill. |
| *Senna* | Wild senna. |
| *Colocynth* | Alum-root. |
| *Tannin* | Smooth sumac. |
| *Olive oil* | Peanut oil. |
| | Beech-nut oil. |
| | Cotton-seed oil. |
| *Laudanum* | Hops. |
| | Mother-wort. |
| *Acacia* | Slippery elm bark. |
| | Sassafras pith. |
| *Bougies* | Slippery elm bark. |

| | |
|---|---|
| *Corks* | Black gum roots. |
| | Tupelo wood. |
| | Corn-cobs. |
| *Allspice* | Spice-bush. |
| *Pink root* | Carindal flower. |
| *Assafœtida* | Wild chamomile. |
| *Calomel* | Dandelion. |
| | Pleurisy root. |
| | Butterfly weed. |
| *Belladonna and Hyoscyamus* | Jamestown weed. |
| *Valerian* | Lady's slipper. |
| *Colchicum* | Indian poke. |

From various physicians, intelligent ladies, and from old Confederate magazines and books and newspapers, I have gathered the following data in reference to peculiar and unusual uses of articles that are incident to our trade, that seemed to be of more or less general employment in the South by physicians, druggists and in Confederate households.

Wood anemone was employed as a vesicatory in removing corns from the feet. Powdered may-apple mixed with resin was used a caustic in treating horses, the farriers using it for escharotic purposes. On the farms the juice of the pulp of the maypop seeds was made into a summer drink in place of lemonade. Powdered blood-root, snuffed up the nose, made a powerful sternutatory and was applied as an escharotic to fungus flesh. Pond-lily poultice was extensively applied to ulcers. Button snakeroot, or globe flower, was used largely as an expectorant and diuretic. Toothache bark (aralia spinosa) was used to allay pain caused by carious teeth, and in South Carolina the negroes relied on it almost exclusively for rattlesnake bite. Side-saddle or flycatcher was used in the various forms of dyspepsia. Ink was made from the rind of the pomegranate fruit and from poke berries. Where during convalescence an astringent tonic was indicated, dogwood supplied the need. This, with the blackberry and gentians and pipsissiwa as tonics and diuretics, and sweet gum and sassafras for mucilaginous and aromatic properties, and wild jalap as a cathartic, supplied the surgeon in camp with easily procurable medicinal plants, which proved sufficient in many times of need.

I here relate another reminiscence of my Alabama soldier friend, Col. Sumter Lea, of Birmingham, using his own language as near as I may be able to repeat it:

"I never heard of but one physician who was promoted on the field. The army once encamped at Tullahoma, Tenn., and obtained their water from a small stream which flowed as well as it could through a dense wood, where the leaves were as thick as in the 'vale of Vallambrosa.' The eddying pools were crystal, bright and clear, but disease and death lurked in their beautiful eddies, for bowel diseases were produced, unusually, among officers and men, and in the absence of any pharmaceutical attachment to the army, it was without remedy until Dr. Cowan, attached as an assistant physician to a Tennessee regiment, adopted the use of what is now the famous 'tablespoon remedy,' consisting of a tablespoon of Epsom salt and equal quantities of bicarbonate soda and laudanum, this dissolved in water and taken a tablespoonful at a dose. This remedy acted magically, and being so widely adopted, attracted the the notice of General Forrest, who, out of admiration and gratitude, promoted Dr. Cowan to his personal staff, with rank of major. There was another doctor who ought to have been promoted for this same sort of service, for diseases of the bowels, during long encampments, became pestilential. The food, especially the bread, when prepared by the ordinary mess soldier, seemed to be especially invented for the production of irritation. Such camp-made biscuit would these days prove a successful rival and threaten the 'rubber trust.'

"An Alabama surgeon named Langhorne, with his hospital assistant, a good-natured fellow called 'Sonk,' grieving over these miseries, determined to find a remedy in his total lack of drugs for these multiplied woes, characterized under the synonyms 'diree' and 'diseremus.' After drawing largely on all their genius, they invented a pill composed of equal parts of red pepper and crude rosin, the latter of which they gathered from the near-by trees and which they consigned to immortality under the name of the 'Diseremus Pill.' It was amusing despite the sadness of the scene, to watch the doctor and his assistant, each with their cup full of their invention, going out to meet the weak and melancholy throng, who in answer to the surgeon's call emerged from their tents, morning after morning, and in single file marched wearily and languidly along, each in turn receiving in his feverish palm a dozen or more of 'Diseremus Pills,' with the laconic instruction to 'take two after each loose operation,' and even these instructions, when the tongue of the doctor grew weary with their constant repetition, was shortened into a sort of ejaculation as the pills were dropped, 'two after each loose,' until this grew into a sort of by-word about the camp."

The bark of the dogwood and swamp willow was mixed with tobacco for smoking. Watermelon juice was made into a syrup, and the rind into preserves. The seed of the watermelon and those of the gourd were used as a diuretic. Gourd rind was used as mould for buttons. The ladies of St. John's Parish, S. C., used prickly pear for hardening tallow in candle making, one pound to four pounds of tallow, taking the place of wax. The hand-leaved violet formed an emollient application. Red maple made an astringent wash.

In the process of dyeing it was found that maple and sweet gum barks with copperas made purple; maple, red oak and copperas, dove color; maple and walnut, brown; sweet gum and copperas, nearly black; peach-tree leaves and alum gave yellow; the artichoke and black oak bark also gave yellow; sassafras root with copperas, a drab; smooth sumac, root and bark and berries, gave black; black-oak bark with a basis of alum gave a bright yellow; with oxide of tin, tints from pale yellow to bright orange; with oxide of iron, a drab; black-oak galls in a solution of vitriol made purple, which as it grows stronger, passed into a black; alum and alder, yellow; hickory bark and copperas, olive; hickory bark and alum, green; white-oak and alum, brown; walnut root and leaves, alone, black; blacksmith's dust was frequently used in place of copperas.

Buckeye lotion was used for gangrenous ulcers, and by some for the toothache.

Among the substitutes for coffee, at home and in camp, the following were a part: Rye, parched okra seeds, cotton seeds, parched sweet potatoes, parched corn hominy, peanuts. It was stated in printed articles "that half the coffee sold in New York and Boston the past twenty-five years has been composed chiefly of rye."

Cotton-seed decoction was used for inflammation in mucous passages. The roots of the cotton plant were employed in asthma, and by the negroes as an abortant. Soap was made from cotton seed by treating them direct with lye.

Among the substitutes for tea were Ceanothus Americanus, known as red root, or New Jersey tea, and holly leaves and blackberry and raspberry leaves and rose leaves.

The Amelia azedarach (China berry) furnished some valuable uses. The berries were employed in making whisky; the bark of the root used as an anthelmintic. The leaves were saved to prevent "botts" in horses, and were used to pack with dried fruits to preserve them from ravages of insects. A soap was made from the berries, called "Poor Man's Soap."

The ox-eyed daisy was used in place of Persian insect powder—an insecticide used as far back as 1857. In the country, fresh elderberry leaves were laid near the head of a bed-ridden person to keep away flies.

In the households and on the farms many interesting expedients were restored to. The newspapers were full of directions about soap-making and for preparing and obtainig the materials. The Richmond *Dispatch* and Wilmington *Journal* published minute directions for making soda from sea weed and corncobs, and recipes for making soaps.

Blackberry wine was used almost exclusively as a substitute for foreign wines, and some wine was also made from wild grapes and the berries of the elder bush. All the newspapers published recipes for making these wines, and there is scarcely a housewife in the South who does not know how to make them to perfection.

In the Mobile *Register* I find the following: "To alleviate the suffering and perhaps save the lives of many of our soldiers, when sickness may be traced to the use of unwholesome water in limestone regions, blackberry cordial is recommended. The following is a good receipt: Bruise the berries and strain through a bag; to each quart of juice add half a pound of loaf sugar, a heaped teaspoonful of powdered cinnamon, the the same of cloves, and a grated nutmeg; boil these twenty minutes, skimming well. When cool, add half pint of brandy for each quart, or add good whisky."

Compound syrup of blackberries was recommended and used as a vehicle for medicines. It was made by adding a half ounce each of cinnamon, allspice, nutmeg, cloves, to half a gallon blackberries. These were boiled twenty minutes in a kettle and strained through a piece of flannel. To this was added loaf sugar to make very sweet, and half pint of cognac brandy to two quarts.

A decoction of the blackberry root and the rind of the pomegranate fruit boiled in milk was a common remedy in diarrhœa.

The roots and leaves of the cockleburr were considered serviceable in passive hemorrhages, diarrhœa, gonorrhœa, and as a deobstruent in obstructions of the spleen and diseases arising from torpid liver.

One or two ounces of a decoction of Indian physic root (Gillonia trifoliata) was given as an emetic, the dose of the powdered root being thirty grains, persisted in until vomiting occurred.

The liquor called *piquette* was largely substituted for cider, wine and beer. It was considered to serve as a tonic, and tended to quench thirst. Directions for making it were as follows: Water was filtered through the pressed and fermented mash of grapes. The mashed grapes were put into a cask, pressed very full, and afterwards hermetically sealed and put in a cool place. When to be used, the head was taken out of the cask, water was added until the whole mass was moistened and water stood on the top. Thus, at the end of the fourth or fifth day the liquor could be drawn off for daily use, the place of the portion used being furnished by a new supply of water. In this way a cask of thirty-six gallons furnished about four gallons of piquette for about twenty days. Piquette was also made from pears, cherries, plums, figs and juniper berries. The rinds of oranges, lemons and aromatic plants, angelica roots, peach leaves, etc., were often added when the drink was too sweet.

Engravers found that the different woods were of hardness as follows: First, the wild currant or service tree and the apple and pear; next, the dogwood, redberry (azalea nudiflora), and kalmia latifolia; then the holly, when well dried; but of all, the boxwood was preferred.

The peach tree furnished a number of uses: The gum was used instead of gum arabic; a tea of the leaves given in whooping cough; the leaves used to season creams instead of vanilla; the leaves used in dyeing.

Beer was made from maize, the persimmon and the sweet locust.

Calycanthus (sweet shrub) was employed as an antispasmodic tonic in case of chronic agues, a strong decoction of the bark of the root or of the seed being given. It was noticed that the root was strongly camphorated.

As an antidote for poison oak the bruised leaves of the Collinsonia canadensis (stone root) were employed; as also the Verbena urticifolia.

Rhus glabra (smooth sumac) was used as a gargle for cleansing the mouth in putrid fevers; and a decoction of the root employed in gonorrhœa and gleet. A vinegar was made from the berries.

Beech-tree leaves, collected in the autumn in dry weather, were used for filling beds, the odor being grateful and they being very elastic.

Black oak was considered efficacious in leucorrhœa, amenorrhœa, chronic hysteria, diarrhœa, rheumatism, cynanche tonsillaris and asthma. The powder of the bark, mixed with lard, was a remedy in painful hemorrhoids, and used as a fomentation in prolapsus uteri and ani, and for deflections in these parts.

I quote from an article of Dr. Daniel Lee, in the *Southern Field and Fireside* of 1860: "It is poor economy for the South to destroy all its valuable tan-bark in clearing oak land, cutting rail timber and firewood, and thereby deprive our descendants of the power to manufacture their own leather. To send a million dollars' worth of hides to the North, have them tanned into leather, made into shoes, boots, saddles and harness for Southern consumption, is to pay about eight million dollars for the support of that Northern economy which never wastes the bark that grows on oak and hemlock trees, and that industry which turns this bark into gold." Such advice as the following was published: "Every farmer ought to save all the tan-bark that he can, for we speak advisedly when we say that the Confederate States are even now short of oak bark if they are to manufacture all the leather they are to consume in saddles, bridles, harness, saddle bags, buggy and carriage harness, caps and hat linings, book bindings, boots and shoes. Since the mechanical trades are essential to our happiness, we should encourage our sons to become scientific mechanics as well as farmers, lawyers, doctors, priests and soldiers."

As substitutes for hemp the following were used: The sunflower stalk, *Asclepias syriaca*, *Urtica diœcia* and *Yucca filamentosa*, or bear grass. The juice of the skin of the blue fig made a red ink. Fig twigs were used as pipe stems. Rope was made of wahoo (*Ulmus alata*), and used in baling cotton. Wax myrtle (*Myrica cerifera*) was employed in making candles, and as a basis for fine soap. The soap was obtained from the berries by boiling and skimming. Four pounds of the wax made forty pounds of the soap, with the other ingredients counted. Candles made by the addition of grease are of a green color. Says the Charleston *Courier* of 1861: "We have been so long dependent on our Yankee enemies for soap and candles that we have forgotten that we can make them ourselves. To our shame, we admit that even on our plantations in the low country and seaboard there are abundant materials for making the best candles in the world, but millions of pounds have been permitted annually to decay unused. The low bush myrtle, indigenous to our coast from Virginia, *ad libitum*, south, the berries of which are now mature, will afford a supply of wax that, with the addition of one-third tallow, will furnish candles sufficient to light every house in the Confederacy for the next year. So, also, on every plantation, nay, in almost every kitchen, the monthly waste of ashes and grease, with the addition of a little lime and salt, and the labor of one person for one day, will make soap enough for our purposes. Now, why should we continue to pay the Yankees 30 cents a pound for soap and 60 cents for candles?" Candles in war time were made of resin. A model, economical candle, sixty yards long, was recommended for the camp and for plantation purposes; it was said to burn six hours a night for six months, and all was at a cost of only a few cents. One pound of beeswax was added to three-fourths pound of rosin, and melted together; four threads of slack twisted cotton was used for a wick, and drawn through the melted wax or rosin three or four times, was wound into a ball, which on pulling the end up and lighting furnished a good candle.

Among the recipes for making soap that were published in the Southern papers, I note the following: 1. Yellow or rosin soap: "Dissolve one pound of concentrated lye in half a gallon of water and three and a half pounds of fat or tallow and boil; put in three-fourths pound of powdered rosin, and let it boil down by constantly stirring until the soap sticks on the kettle and gets very thick. Put into a mould. 2. Hard fancy soap: Dissolve half pound of concentrated lye in two and a half pounds of hot water, and let cool; then melt by a low heat five pounds of clear fat or tallow; pour in the lye in a very small stream, and stir rapidly. Keep stirring until all has assumed the appearance of thick honey. Let it stand for twenty-four hours, when it will have set into a fine hard soap, which may be perfumed or variegated with colors by stirring in the desired perfume or coloring matter, just before covering. 3. Soft soap: One pound concentrated lye and three gallons soft water and five pounds of fat or tallow. Boil until the mass grows transparent and all the fat has disappeared. Add fifteen gallons of water and boil a few minutes, and the soap will be ready for use.

In making gunpowder the lighter woods, such as willow, dogwood and alder charcoal, were recommended. I append an advertisement taken from the Augusta, Ga., *Chronicle* of 1862: "To Contractors—Willow wood wanted! 500 cords willow will be contracted for, to be delivered on the line of the canal at the government powder factory at Augusta, Ga., at the rate of not less than 100 cords a month, commencing 1st December next."

Out of the wood of the white poplar, split into shavings like tape or braid, the stuff called *sparterie* was made, used in the manufacture of hats. It was said that one workman with the aid of a child to carry off the shavings could keep a half dozen plaiters employed.

Shoes were made from canvas for uppers and tupelo wood for soles, for the negroes on the plantations. They had been patented, so it was said, by Henry Wyatt & Co., of New York, who offered wooded soled brogans for the negroes of the South. Ropes and baskets were made from the bark of the Canada leatherwood.

The following was published concerning the sassafras tree: "The sassafras wood stripped of its bark is very durable and strong, resists worms, etc. It forms an excellent post for gates. Bedsteads made of it are never infested with bugs. The pith of the young shoots and the leaves contain much mucilage and are used extensively in New Orleans to thicken pottage and in making the celebrated 'gumbo' soup.

"A cheap and wholesome beer for soldiers, or as a table beer, is prepared from the sassafras. Take eight bottles of water, one quart molasses, one pint yeast, one tablespoon of ginger, and one and a half tablespoons of cream tartar; mix and stir in an open vessel after standing twenty-four hours."

As far back as 1857 it was suggested in the patent office reports (says a Confederate publication), that the Pyrethrum would be found to answer the purpose of destroying insects, lice, etc., on plants and animals, and up to now, as far as I know, this has not been sufficiently experimented with.

W. Gilmore Sims wrote a friend that, "the persimmon beer made in Orangeburg, Dist., S. C., by Hon. J. M. Felder, equalled the best sparkling 'Jersey champagne,' or a carbonated cider. The old Southern song ran: 'Christmas comes but once a year, eggnog and 'simmon beer.'" It was customary to mash the fruit, strain through a coarse sieve, knead with wheat bran and bake in an oven. This persimmon bread could be put away for winter use in making beer when wanted.

Cloth was rendered waterproof, according to a published recipe, by adding to one gallon of turpentine two and a half pounds of beeswax. Boil well in a pot, remove from the fire, and while it is hot put in the goods. Saturate well and hang up to dry. Use one gallon of turpentine to eight yards of cloth.

A correspondent in the Charleston *Mercury* wrote from Waresboro, Ga.: "You speak of black moss for mattresses. Our common palmetto leaves, split into shreds with fork and hackle, boiled, dried in the sun a few days, make a light, clean, healthy and durable mattress. Let me suggest that palmetto pillows would be light and comfortable for our soldiers on the coast. Their corn and flour sacks, in the absence of anything better, furnished ready made pillow ticks. Our negroes are busily employed making light, durable and handsome palmetto hats for our soldiers. A bed made from the downy swamp plant which our people call 'cat's tail' took a premium at a late agricultural fair in South Carolina."

I enumerate a few more medicinal uses that were made of some of the prodncts of our Southern fields and forests by our physicians and housewives, and will close.

Phytolacca decandra, or poke, was largely used in diseases affecting the scalp, and in ulcers, irruptions, itch and hemorrhoids. Knot grass was considered a powerful astringent in diarrhœa and uterine hemorrhages. Water pepper, says a writer at Manchester, S. C., was used in his family in 1862 in dysentery, and every case was improved and cured. Mountain laurel was employed with claimed success in rheumatism, gout and glandular enlargements. Black alder used as wash in cutaneous troubles. Holly leaves used as an emetic, and birdlime made from the middle bark. Love vine used as a laxative tea. Pinckneya pubens, Georgia bark, useful in intermittent fevers. It is said that "Dr. Fair detected a considerable amount of cinchonine it it, but was prevented from continuing his examination."

Woodbine was given in asthma, and a decoction of the flowers administered to calm the pain of colic following childbirth. A decoction made by pouring boiling water over the leaves, flowers or berries of the elder bush was used as a wash for wounds to prevent injuries from flies. Sea myrtle was used in popular practice in South Carolina as a pallative and demulcent in consumption and coughs, a strong decoction given several times a day. Raw-weed used in whisky in place of quinine in Maryland. Cat weed employed in popular practice in diseases of chest and bowels. Hound's tongue employed in domestic practice as a mucilaginous drink, and the roots made into poultice in case of bruises, sprains, etc. Gravel root given as an emetic. Horse nettle used as an aphrodisiac among the negroes. Virginian silk used as a diuretic decoction in gonorrhœa. The buds and inside bark of the long-leaved pine and bits of pine steeped in gin were favorite domestic remedies in coughs and colds, and as a diuretic.

What I have here collected has been put together in a busy season and during the war excitements that have just been engaging the attention of all our people. The result is not intended as a complete history of the conditions named. It could, necessarily, only be a part of the history of those conditions.

In designing this paper, I had hoped to make it more complete by using contributions from surgeons of the Confederate army and navy, and druggists engaged in business during the period, so far as they were living and from papers to be loaned me by them. Out of scores of letters addressed to living men of this character, I received but few replies. In obtaining some of the matter, railway trips had to be taken, and much of it was collected at considerable expense and labor. When it is remembered that the conditions that are suggested here lasted for a period of nearly four years, then the sufferings and the achievements and the heroism of seven millions of people are in a measure made manifest.

If I have succeeded in recalling by way of suggestion some facts that in the present may be of use, or in the future may be evolved into utility, I will have been rewarded for my outlay and my efforts.

The war of 1861–1865 is now but a memory. The heroes of both sides—those "tented" on "fame's eternal camping ground" and the survivors—are now dear to a reunited people, who, proud of the common victories of their fellow-countrymen at Manila and Santiago, and rejoicing in the vigor of American arms and the glory of American ideals, stand expectantly awaiting and hopefully facing the great future in store.

